# Cognitive Deficit in Heart Failure and the Benefits of Aerobic Physical Activity

**DOI:** 10.5935/abc.20180002

**Published:** 2018-01

**Authors:** Maria Luíza de Medeiros Rêgo, Daniel Aranha Rego Cabral, Eduardo Bodnariuc Fontes

**Affiliations:** Universidade Federal do Rio Grande do Norte (UFRN); NeuroEx, Natal, RN - Brazil

**Keywords:** Heart Failure, Indicators of Morbidity and Mortality, Cognition, Exercise

## Abstract

Heart Failure is a clinical syndrome prevalent throughout the world and a major contribution to mortality of cardiac patients in Brazil. In addition, this pathology is strongly related to cerebral dysfunction, with a high prevalence of cognitive impairment. Many mechanisms may be related to cognitive loss, such as cerebral hypoperfusion, atrophy and loss of gray matter of the brain, and dysfunction of the autonomic nervous system. The literature is clear regarding the benefits of aerobic physical activity in healthy populations in the modulation of the autonomic nervous system and in brain functions. Studies have shown that in the population of patients with heart failure, exercise is associated with an improvement in cognitive function, as well as in cardiac autonomic regulation. However, little emphasis has been given to the mechanisms by which aerobic physical activity can benefit brain functioning, the autonomic nervous system and result in better cognitive performance, particularly in patients with heart failure. Therefore, the present work presents the ways in which brain areas responsible for cognition also act in the modulation of the autonomic nervous system, and emphasizes its importance for the understanding of cognitive impairment in relation to the pathophysiology of heart failure. It is also described the way in which aerobic physical activity can promote benefits when it is integrated into the therapy, associated to a better prognosis of the clinical picture of these patients.

Heart Failure (HF) accounts for about 50% of all hospitalizations occurring in South America^1^ and is one of the most frequent causes of hospitalization for cardiovascular diseases.^2^ In addition to the direct influence on cardiac autonomic control, HF is strongly related to the presence of cerebral dysfunction and cognitive impairment, affecting approximately 75% of this population.^3^ This cognitive deficit is associated with executive functions, including difficulties in the planning and execution of actions, low ability to solve problems and inhibit behaviors.^4^ In practice, this results into less ability to perform daily activities such as shopping, feeding and locomotion - including walking - in addition to being related to lower self-care levels, higher hospitalization rates, increased expenses with more frequent hospitalizations, and, finally, there is an increase in morbidity and mortality in this pathology. In this sense, several treatments are performed in order to mitigate the deleterious effects caused by HF. However, such treatments usually involve invasive and / or medicamentous procedures such as heart transplantation, left ventricular assist device, beta-blockers, aldosterone antagonists, and angiotensin converting enzyme inhibitors. All these drugs, despite having proven beneficial results, can develop several types of side effects such as renal failure and hyperpotassemia.^2^ In this sense, physical exercise has been pointed out as an important auxiliary tool in the treatment of patients with HF, however, little has been analyzed about its benefits to brain function. In the present work, the pathways by which the prefrontal cortex (PFC) is closely linked to the regulation of cardiac autonomic control and its influence on cognitive impairment in HF patients are presented. In addition, it is described how the regular practice of physical activity can promote benefits to brain function and cognitive performance in this population, as well as the contribution on cardiac autonomic control already widely described.

In the search for the genesis of this problem, many mechanisms may be related to cognitive loss, such as cerebral hypoperfusion, atrophy and loss of gray matter of the brain, as well as autonomic nervous system (ANS) dysfunction.^5^ A neuroimaging study in FC II patients found that individuals with this syndrome had impairment in several brain areas such as the hippocampus (short-term memory conversion in long-term memory), caudate nucleus (modulation of body movements), PFC (executive functions: decision-making, planning, inhibitory control) and hypothalamus, fundamental areas in cognitive processes and autonomic control.^5^ In this perspective, it is worth mentioning the existence of a recent pathophysiological model of cognitive decline in this population, which states that a set of factors such as hypoperfusion, hypoxia, inflammatory cytokines increase, thromboembolic diseases and hemodynamic abnormalities can lead to brain mass atrophy, generating cognitive deficits.^6^ Another important point to emphasize about the pathophysiology imposed by HF is the severe dysfunction in ANS, characterized by increased sympathetic tone and decreased parasympathetic^7^ and may be related to vasoconstriction.^8^ As a consequence of this autonomic balance with sympathetic overlap, there is difficulty in the arrival of blood in various systems of the body, including the brain. Cerebral hypoperfusion in patients with HF may lead to reduced functional capacity^9^ and cognitive deficits.^5^ More specifically, permanent impairment of cerebral perfusion and chronic ischemia in deep areas may result in cognitive impairment and difficulty performing routine activities. The hippocampus, for example, has neural plasticity in front of the lower supply of oxygen and may have decreased mass and possibly cause memory impairment.^10^

It has been demonstrated that FC I-III patients have low cerebral blood flow (CBF) in the sitting position and this is related to low cardiac output, which can cause intense hypoperfusion in the accomplishment of simple tasks, such as the act of leaving a position lying to a sitting.^9^ Therefore, with less supply of oxygen to the brain, cognitive impairments are virtually unavoidable. In addition, it has already been observed that the degree of cognitive dysfunction may serve as a predictor of cardiovascular complications in patients with HF. In the study, 246 FC II - IV patients performed the Montreal Cognitive Assessment to evaluate cognitive ability and it was shown that the worst performance in the test was those with the greatest possibilities of cardiovascular events in up to 180 days.^11^ Thus, cognition seems to be closely related to the degree of cerebral oxygenation, and the more hypoperfused the brain, the greater the dysfunction of the nervous system, indicating a greater impairment in cardiac output and a lower ability to modulate autonomic activity, increasing the risk of cardiovascular and cerebrovascular events.

Literature is clear on the benefits of aerobic physical activity (APA) in healthy populations, on SNA modulation, and on brain and cognitive functions. Evidence shows that chronically performed APA has the potential to promote beneficial effects on cardiovascular health, mediated by increased vagal tone and decreased sympathetic activity in the sinus node, with improved vascular function, cardiac remodeling, and renal-adrenal functions.^12^ However, regular APA has also demonstrated an important role in the modulation of some brain regions of fundamental importance in cognitive processes, through the increase of CBF in PFC,^13^ increased volume in the hippocampus,^10^ increased concentrations of vascular endothelial growth factors (VEGF) (new capillary growth) and brain-derived neurotrophic factor (BDNF) (strengthening of synaptic connections)^14^, as well as angiogenesis in lobofrontal regions.^15^ Therefore, these functional, biochemical, and morphological changes in the brain are strongly linked to cognitive improvement.

Following this line of reasoning, in a study with FC III patients and ejection fraction ≤ 35%, an 18 week intervention (twice a week) of physical exercises was performed alternating between treadmill, cycle ergometer and stair simulator. The results demonstrated that exercise improved the cognitive functions of selective attention and psychomotor speed.^16^ In addition, the performance of the six-minute walk test and the Mini Mental State Exam score have been directly correlated, indicating that the lower the functional cardiovascular capacity of the subject, the lower their cognitive ability.^17^ In the meantime, it is worth mentioning that patients usually leave the doctors' offices having heard that the practice of physical exercises is important to combat the sedentary lifestyle, improve the function of the heart muscle and, therefore, become healthier and with a better quality of life. However, the low emphasis on cognitive benefits and the improvement in brain function that physical activity can provide for HF patients is of concern.

In this context, a recent review addresses the practice of APA as a beneficial factor in preventing and even reversing the cognitive impairment caused mainly by the decrease in CBF in this population. This benefit would be a consequence of both the improvement in cardiac muscle contractile activity and the decrease in peripheral vascular resistance due to a decrease in sympathetic activation.^18^ Furthermore, the possible effects of APA on the ANS of patients with heart disease have been demonstrated, as in the case of HF, indicating the possible sympatovagal modulation with increased parasympathetic tone and a decrease in sympathetic activity, which is an extremely important and decisive clinical condition for this population.^19^ However, little emphasis was given to the integration between the CBF and the autonomic nervous system, as well as in the modulation of the ANS as a result of exercise in patients with HF, besides the possible integration pathways resulting from this modulation of the ANS and its influence on activity in general, implying greater benefits for the individual.

The pathways through which frontal regions, such as the PFC, act in the modulation of the ANS are important for the understanding of the pathophysiology of HF, as well as to understand how the APA can interfere in these two aspects (cognition and cardiac autonomic control). In this sense, a neurovisceral model was proposed by which the PFC has an inhibitory function on the amygdala^20^ (an integrative area that receives sensory afferents, confers an emotional characteristic and emits eferences to cortical areas). However, when this region is uninhibited, a situation that can occur due to the lower CBF in the PFC, allows the activation of sympathetic neurons and a decrease in the action of parasympathetic neurons, both in the brainstem^20^, triggering a nervous autonomic balance with sympathetic predominance. It is known that APA plays an active role, especially in CPF^13^ and autonomic modulation^12^, creating a feedback system in which APA acts in several spheres, culminating in the cognitive improvement of patients (Figure 1) and, consequently, improving the prognosis.

**Figure 1:**
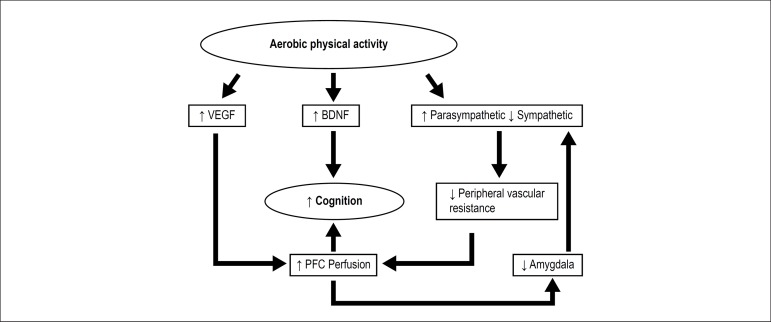
Aerobic physical activity promotes an increase in the concentrations of VEGF and BDNF, which can improve cognitive processes, increase the parasympathetic tone, and decrease the sympathetic activation. This condition may decrease peripheral vascular resistance and lead to increased cerebral blood flow in the prefrontal cortex, positively interfering with cognitive ability. With increased cerebral blood flow in the prefrontal cortex, there may be an inhibition of the amygdala promoting a vagal increase and sympathetic decrease, feedback system. VEGF: vascular endothelial growth factor; BDNF: brain-derived neurotrophic factor

Thus, it can be observed that the damages caused in the cognitive aspects are often reversible and, therefore, possible to improve the executive functions,^18^ increasing the quality of life of these patients. It is worth mentioning, then, that APA is a useful tool as part of the treatment of these patients, besides being a non-pharmacological alternative and low cost. Although APA is easy to perform in healthy subjects, the same cannot be said for patients with HF. FC IV patients, for example, are not advised to perform physical activity. In addition, some procedures should be done before the practice, such as performing the maximum effort test for physical and clinical condition analysis. The prescription should be made based on evaluations performed periodically by the cardiologist and on the risk stratification of the patient and the practice should not be performed without supervision.^2^

Therefore, the importance of physical activity in a much broader context that goes beyond the improvement of the heart, which is the benefit of the brain function of patients with heart failure, through functional and morphological changes in the brain and ANS, is clear, implying greater efficiency in cognitive processes. Therefore, it is valid to emphasize once again the prescription of APA with a focus on cognition, with the justification of enhancing performance in the basic and instrumental activities of this population and explaining its practical benefits and its contribution to the possibility of greater well-being of the patients during the evolution of the clinical condition.

Finally, in addition to combating sedentary lifestyle and improved heart muscle, APA prescription should be focused on cognitive benefit, with repercussion in the management of possible daily limitations, such as shopping, compromising understanding, communication and interpersonal relationships. This can be a way to increase the patient's adherence to this therapeutic component, aiding in his treatment and bringing more benefits and quality of life.
